# UPLC-Q-TOF/MS-based metabonomic studies on the intervention effects of aspirin eugenol ester in atherosclerosis hamsters

**DOI:** 10.1038/s41598-017-11422-7

**Published:** 2017-09-05

**Authors:** Ning Ma, Yajun Yang, Xiwang Liu, Xiaojun Kong, Shihong Li, Zhe Qin, Zenghua Jiao, Jianyong Li

**Affiliations:** Key Lab of New Animal Drug Project of Gansu Province, Key Lab of Veterinary Pharmaceutical Development, Ministry of Agriculture, Lanzhou Institute of Husbandry and Pharmaceutical Science of Chinese Academy of Agricultural Sciences, Lanzhou, China

## Abstract

Based on the pro-drug principle, aspirin and eugenol were used to synthesize aspirin eugenol ester (AEE) by esterification reaction. In present study, the anti-atherosclerosis effects of AEE were investigated in hamsters with the utilization of metabonomic approach based on UPLC-Q-TOF/MS. Biochemical parameters and histopathological injures in stomach, liver and aorta were evaluated. In atherosclerotic hamster, oral administration of AEE normalized biochemical profile such as reducing TG, TCH and LDL, and significantly reduced body weight gain, alleviated hepatic steatosis and improved pathological lesions in aorta. Slight damages in stomach mucous were found in AEE group. Plasma and urine samples in control, model and AEE groups were scattered in the partial least squares-discriminate analysis (PLS-DA) score plots. Thirteen endogenous metabolites in plasma such as lysophosphatidylcholine (LysoPC), leucine and valine, and seventeen endogenous metabolites in urine such as citric acid, phenol sulphate and phenylacetylglycine were selected as potential biomarkers associated with atherosclerosis. They were considered to be in response to anti-atherosclerosis effects of AEE, mainly involved in glycerophospholipid metabolism, amino acid metabolism and energy metabolism. This study extended the understanding of endogenous alterations of atherosclerosis and offered insights into the pharmacodynamic activity of AEE.

## Introduction

As a growing health challenge in the world, atherosclerosis is a complex chronic disease characterized by the accumulation of lipids within arterial walls, dyslipidemia, endothelial dysfunction, and chronic inflammation^1^. Atherosclerosis can cause narrowing, hardening and even complete blockage of arteries, which is a major cause of mortality in patients with cardiovascular diseases (CVD). Eugenol is a light yellowish oily liquid extracted from certain essential oils such as clove oil, nutmeg, cinnamon, basil and bay leaf. Evidences from numerous studies have showed that eugenol possesses antibacterial, antiviral, antifungal, antioxidant, antithrombotic, antiparasitic, and anti-inflammatory properties^2–5^. In addition, eugenol could lower blood lipids to alleviate hyperlipemia, which is beneficial to the inhibitation of atherosclerosis^6–8^. Therefore, eugenol has gained the attraction from researchers in atherosclerosis prevention and treatment. However, irritation and vulnerablity to oxidation are the main constraints in its application, which are mainly caused by the phenolic hydroxyl group.

In ancient China and Egypt, natural substance salicylic acid and its derivatives obtained from willow bark or leaves had been used to ease pain, fever and inflammation. Acetylsalicylic acid (aspirin), the best-known salicylic derivative, was synthesized by Felix Hoffman in 1897^9^. In addition to the anti-inflammatory, antipyretic and analgesic effects, aspirin is widely used to reduce the risk of CVD and certain cancers^10, 11^. Moreover, increasing reports have demonstrated that aspirin has therapeutic effects on atherosclerosis^12, 13^. However, the side effects of aspirin such as gastrointestinal damage limit its application^14^.

In order to increase the therapeutic effects of aspirin and eugenol on atherosclerosis and reduce their disadvantages, aspirin eugenol ester (AEE) was synthesized according to pro-drug principle^15^. AEE is a white and odorless crystal. In the chemical structure of AEE, carboxylic group from aspirin and hydroxyl group from eugenol were chemically masked to reduce the gastrointestinal side effects and improve structural stability^16^. The toxicological studies indicated that AEE was non-genotoxic *in vitro* or *in vivo* and its toxicity was obvious lower than its precursors, which suggested that AEE was a safe compound with good druggability^17, 18^. Metabolism study proved that AEE was decomposed into salicylic acid and eugenol after administration, which could show their original activities and act synergistically^19^. Moreover, pharmacodynamics experiments showed AEE could regulate blood lipid levels in hyperlipidemic rats such as reducing triglycerides (TG), total cholesterol (TCH) and low density lipoprotein cholesterol (LDL)^20, 21^. However, no data has been reported the effects of AEE on atherosclerosis.

With the comprehensive analysis of small molecules, metabonomics is a versatile tool to evaluate the toxicity or therapeutic effect of compounds from the understanding of the dynamic biochemical compositions. In present study, the atherosclerosis in Syrian golden hamster induced with high fat diet (HFD), an excellent model for studying atherosclerosis^22^, was used to evaluate the effects of AEE on atherosclerosis with the application of UPLC-Q-TOF/MS-based plasma and urine metabonomics. Meanwhile, the body weight gain (BWG), blood biochemical indices, atherosclerosis index (AI) and the histopathological changes of aorta, stomach, and liver were also investigated in the study.

## Materials and Methods

### Chemicals and reagents

AEE (transparent crystal, purity: 99.5% with RP-HPLC) was prepared in Lanzhou Institute of Husbandry and Pharmaceutical Sciences of Chinese Academy of Agricultural Science. MS-grade formic acid was supplied by TCI (Shanghai, China). Deionized water (18 MΩ) was prepared with a Direct-Q®3 system (Millipore, USA). MS-grade acetonitrile was purchased from Thermo Fisher Scientific (USA). Carboxymethylcellulose sodium (CMC-Na) was supplied by Tianjin Chemical Reagent Company (Tianjin, China).

### Animal experiment

A total of 30 male Syrian golden hamsters weighing 100–110 g were purchased from Charles River Company (Vital River, Beijing, China). All animals were housed in facilities by group at a controlled relative humidity (45–65%) and temperature (22 ± 2 °C). Hamster feed and drinking water were supplied *ad libitum*. All of the experimental protocols and procedures were approved by the Institutional Animal Care and Use Committee of Lanzhou Institute of Husbandry and Pharmaceutical Science of Chinese Academy of Agricultural Sciences (Approval No. NKMYD201601). Animal welfare and experimental procedures were performed strictly in accordance with the Guidelines for the Care and Use of Laboratory Animals issued by the US National Institutes of Health.

### Study design

**Figure 1:**
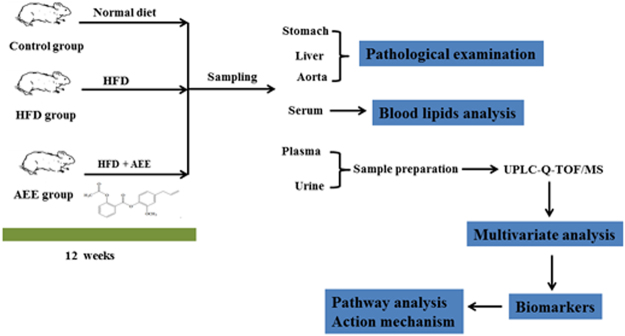
Schematic overview of the experiment design.

The experimental design was shown in Fig. 1. Hamsters were assigned into 3 groups (n = 10): (1) control group, in which hamsters were fed with normal diet; (2) high fat diet (HFD) group, in which hamsters were fed with HFD; (3) AEE group, in which the hamsters were simultaneously fed with HDF and AEE (27 mg/kg body weight). The normal diet (12.3% lipids, 63.3% carbohydrates and 24.4% proteins) was purchased from Keao Xieli Feed Co., Ltd (Beijing, China) and the atherogenic HFD (40% lipids, 43% carbohydrates and 17% proteins) was supplied by Research Diet, Inc. (product D12079B, New Brunswick, NJ).

In the subchronic toxicity, the no-observed-adverse-effect level (NOAEL) of AEE was considered to be 50 mg/kg/day^18^. Meanwhile, in our previous study, five week treatment of AEE dosed at 18, 36, 54 mg/kg can reduce the levels of TG, TCH and LDL in hyperlipidemic rats^23^. Based on the dose used in the former studies, the dose of AEE was selected as 27 mg/kg in the present study. The study was conducted for 12 weeks and AEE suspensions were prepared in 0.5% CMC-Na. According to the individual body weight, hamsters in AEE group were intragastrically (i.g.) administered with AEE. For eliminating the effect of CMC-Na (vehicle), hamsters in control and HFD groups were treated with equal volume of CMC-Na as AEE group.

### Sample collection

After fasted for 10–12 hours, hamsters from each treatment group were sacrificed under anesthesia induced with pentobarbital (intraperitoneal injection, 30 mg/kg). Blood samples were collected from the heart into normal and heparin-treated vacuum tubes to prepare serum and plasma, respectively. Serum and plasma were obtained after centrifugation of blood (2500 rpm at 4 °C for 10 min), and stored at −80 °C until analysis. Individual hamsters were placed in metabolic cages (1 per cage) to obtain 24-hour urine collections and be stored at −80 °C before analysis. The aorta was carefully isolated from hamster and extravascular fat tissue was removed. The tissues of aorta, liver and stomach were subsequently fixed in 4% formalin for pathological observations.

### Measurement of biochemical parameters

Serum was analyzed using an automatic biochemistry analyzer (Erba XL-640, German). Biochemical parameters including total bilirubin (T-BIL), total protein (TP), albumin (ALB), alanine transaminase (ALT), aspartate aminotransferase (AST), lactate dehydrogenase (LDH), glucose (GLU), triglycerides (TG) and total cholesterol (TCH), low density lipoprotein cholesterol (LDL) and high density lipoprotein cholesterol (HDL), were analyzed in the experiment. The AI was calculated as followed: AI = (TCH - HDL)/HDL^24^. The kits for biochemistry analysis were provided by Ningbo Medical System Biotechnology Co., Ltd (Ningbo, China).

### Histopathological examination

In order to investigate the histopathological changes, tissues of aorta, liver and stomach were formalin-fixed and paraffin embedded, sectioned, and stained with hematoxylin and eosin (HE) by using the standard protocol^25^. HE-stained sections were examined using a 13395H2X microscope (Leica, Germany). The morphometric analysis of the aorta images was carried out by Image-Pro Plus 6.0 software (Media Cybernetics, Bethesda, MD, USA). The stenosis ratio was calculated as percent of lumen area to total area of the aorta as previously described^26^.

### Sample preparation

The plasma samples were thawed at room temperature prior to analysis. Acetonitrile (400 μL) was added to every 200 μL plasma. After vigorous vortex-mixing for 1 min and incubation for 10 min, the mixture was centrifuged at 12,000 *g* for 15 min at 4 °C to precipitate the proteins. The supernatant was filtered through a 0.22 μm nylon filter. An aliquot of 3 μL sample was injected for analysis. Urine samples were thawed at room temperature, and then 600 μL ice-cold methanol was added into 200 μL of urine, vortex mixed and centrifuged at 13,000 g for 15 min at 4 °C to remove solid materials. The supernatant was also filtered through a 0.22 μm nylon filter and an aliquot of 4 μL was injected for analysis.

### Data acquisition and processing

Metabonomics analysis was performed with an Agilent 1290 Infinity LC system coupled to an Agilent 6530 Accurate-mass Q-TOF mass spectrometer (Agilent, USA). Chromatographic separations of plasma and urine samples were performed on an Agilent ZORBAX SB-C18 threaded column (2.1 × 150 mm, 1.8 μm, Agilent Technologies, USA) maintained at 35 °C. The mobile phase consisted of solvent A-water with 0.1% formic acid and solvent B-acetonitrile with 0.1% formic acid. The optimized gradient program was shown in Table S1. Flow rate of plasma sample was 0.3 mL/min, and 0.35 mL/min of urine sample. The post time was set to 5 min for equilibration. Mass spectrometry was performed both in electrospray ionization in positive (ESI+) and negative (ESI−) ion modes. The fragment voltage was set at 135 V and skimmer voltage was set at 65 V. The capillary voltages were set at 4.0 KV in positive mode and 3.5 KV in negative mode, respectively. The drying gas flow (nitrogen) was set to 10 L/min at 350 °C and the nebulizer pressure was set at 45 psig. Data was collected in centroid mode from 50–1000 m/z using an extended dynamic model.

The raw MS data were firstly processed by Mass Hunter Qualitative Analysis software (Agilent technologies, USA) to converted to common data format (.mzData). The program XCMS was used for nonlinear alignment of the data in the time domain and automatic integration and extraction of the peak intensities. The parameters of the XCMS were default settings. The data were filtered by interquantile range and normalized to the total intensity for further multivariate data analysis. The obtained data were imported into SIMCA-P (version 13.0, Umetrics AB, Sweden) where principal component analysis (PCA) and partial least squares discriminant analysis (PLS-DA) were performed to data set analysis. The quality of PLS-DA models was described by R^2^X, R^2^Y, and Q^2^ and its validity was evaluated by permutation testing (with 200 permutations). Variable importance in the projection (VIP > 1) value of validated PLS-DA model and the *P* values of one-way ANOVA (*P* < 0.05) were taken as the measurement indices for potential metabolites selecting. Identification of the metabolites was achieved through a mass-based search followed by manual verification. TOF-MS accurate mass value of the molecular ion of interest was searched against the METLIN and Human Metabolome Database (HMDB). Then, MS/MS analysis was carry out to confirm the structure of potential biomarkers by matching the masses of the fragments. The clustering analysis of the potential biomarkers and pathway analysis were performed on MetaboAnalyst 3.0 (http://www.metaboanalyst.ca/), and the metabolic pathway interpretation was performed using the KEGG database.

### Statistical analysis

BWG, AI and blood biochemical parameters were expressed as mean ± standard deviation (SD). The differences had been evaluated by one-way ANOVA with Fisher’s least significant difference (LSD) test using the Statistical Package for Social Science program (SPSS 16.0, Chicago, IL, USA). Differences were considered significant at *P* < 0.05.

## Results

### AEE reduced body weight gain

**Figure 2:**
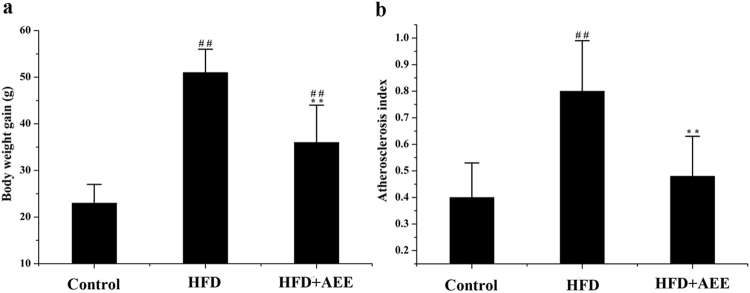
Effects of AEE on the body weight gain (**a**) and atherosclerosis index (**b**) in atherosclerosis hamsters. Data were expressed as mean ± standard deviation. HFD: high fat diet group; ^##^ *P* < 0.01 significant difference from the control group; ^**^ *P* < 0.01 significant difference from HFD group.

Before the experiment, no difference was observed in body weight among the groups. Body weight gains (BWGs) of hamsters in different treatment groups were analyzed at the end of the study (Fig. 2a). After feeding with HFD for 12 weeks, the hamsters in HFD group had higher BWGs than that in the control (*P* < 0.01). In comparison with the HFD group, BWGs were significantly decreased in AEE group (*P* < 0.01). Significant difference was found between control and AEE groups (*P* < 0.01), indicating the elevated BWGs induced by HFD were partly recovered by AEE treatment.

### AEE ameliorated biochemical profile disorder

The results of serum biochemical parameters were shown in Table 1. After feeding with HFD for 12 weeks, the levels of biochemical parameters were all significantly increased in the serum of the HFD group than that in the control group (*P* < 0.01). Compared with the HFD group, after AEE treatment, the levels of AST, HDL, LDL, TCH, TBIL, TP, LDH, ALB, GLU and TG were significantly reduced (*P* < 0.01). No significant difference was observed in ALT between HFD and AEE groups. Biochemical parameters in AEE group including TBIL, TP, AST, LDH, TG, LDL and TCH showed no difference when compared with the control. However, the levels of ALB, ALT, GLU and HDL in AEE group were significant higher than those in the control (*P* < 0.01). These results suggested that AEE reversed the disturbance in biochemical profile caused by HFD. According to the values of TCH, HDL and LDL, the AI was calculated in the study (Fig. 2b). The AI was markedly elevated in the HFD group in comparison with the control (*P* < 0.01). In AEE group, the AI values were significantly reduced than those in the HFD group (*P* < 0.01), and no statistical difference was observed between control group and AEE group, indicating the blood lipid levels were improved in hamsters with AEE treatment.

**Table 1:** Effects of AEE on serum biochemical indices in different treatment groups.

Variables	Control	HFD	AEE
TBIL (umol/L)	1.1 ± 0.2	1.9 ± 0.6^##^	1.1 ± 0.3^**^
TP (g/L)	38 ± 5	50 ± 8^##^	40 ± 4^**^
ALB (g/L)	18 ± 3	28 ± 4^##^	23 ± 3^**##^
ALT (U/L)	48 ± 8	70 ± 15^##^	64 ± 12^##^
AST (U/L)	70 ± 15	107 ± 25^##^	59 ± 17^**^
LDH (U/L)	143 ± 40	222 ± 49^##^	119 ± 27^**^
GLU (mmol/L)	5.9 ± 1.2	10.2 ± 2.0^##^	8.9 ± 1.8^**##^
TG (mmol/L)	1.41 ± 0.35	2.71 ± 0.50^##^	1.09 ± 0.28^**^
HDL (mmol/L)	0.99 ± 0.22	1.96 ± 0.06^##^	1.27 ± 0.21^**##^
LDL (mmol/L)	0.32 ± 0.09	0.61 ± 0.12^##^	0.29 ± 0.08^**^
TCH (mmol/L)	1.73 ± 0.37	3.48 ± 0.52^##^	1.6 ± 0.43^**^

HFD: high fat diet; AEE: aspirin eugenol ester; ^##^ *P* < 0.01 significant difference from control group; ^**^ *P* < 0.01 significant difference from HFD.

### AEE inhibited liver and atherosclerotic lesions

**Figure 3:**
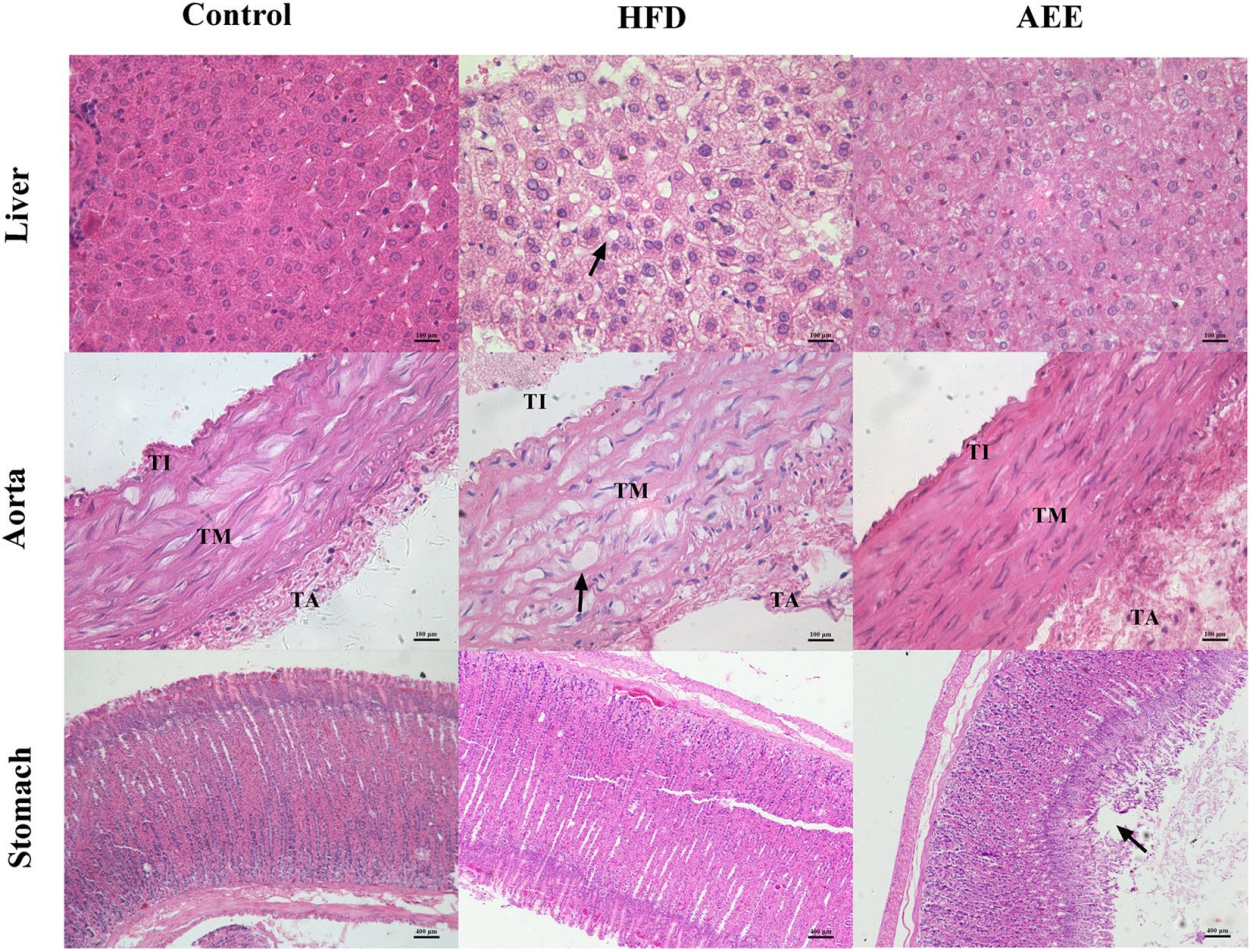
Representative photographs of hematoxylin–eosin (HE) staining of liver (×400), aorta (×400) and stomach (×100) in different treatment groups. TA: tunic adventitia; TM: tunic media; TI: tunic intima. Compared with the liver in control hamster, large fat droplets were observed in the liver of the hamsters fed with high fat diet; The structure of blood vessel in control group was integrated and the TI was smooth, whereas lots of foam cells, migration of smooth muscle cells and serious accumulation of fat were observed in HFD group; Hepatic steatosis and pathophysiologic changes of aorta were notably alleviated by AEE treatment. The slight necrosis and ecclasis of gastric mucosa were found in the AEE group. The typical pathological changes of liver, aorta and stomach were indicated by black arrows, respectively.

The results of the histopathological changes in the different groups were illustrated in Fig. 3. Histopathological examination of the liver in the control group showed that hepatocyte of hamster was normal and nuclear structure was clear, while significant morphological changes were observed in the HFD group. In the HFD group, liver sections showed that the hepatocyte had a large area with hydropic degeneration and some were found with cytolysis and fatty degenerations. In AEE treatment group, hepatocyte was nearly normal with the reduction of hydropic and fatty degenerations. From the above results, HFD consumption stimulated fat accumulation in hepatic cells and finally caused fatty liver in the atherosclerosis hamsters, whereas AEE reversed the HFD-induced liver injury. These results were in accordance with the results of serum biochemical parameters.

Gastrointestinal effects of oral administration of AEE were examined in the study. No damaging effects on the stomach were observed in control and HFD groups. After oral administration of AEE dosed at 27 mg/kg for 12 weeks, stomach mucous membrane of hamster became uneven, and the evidence of slight degeneration, necrosis and ecclasis of mucosa epithelium could be found (Fig. 3).

**Figure 4:**
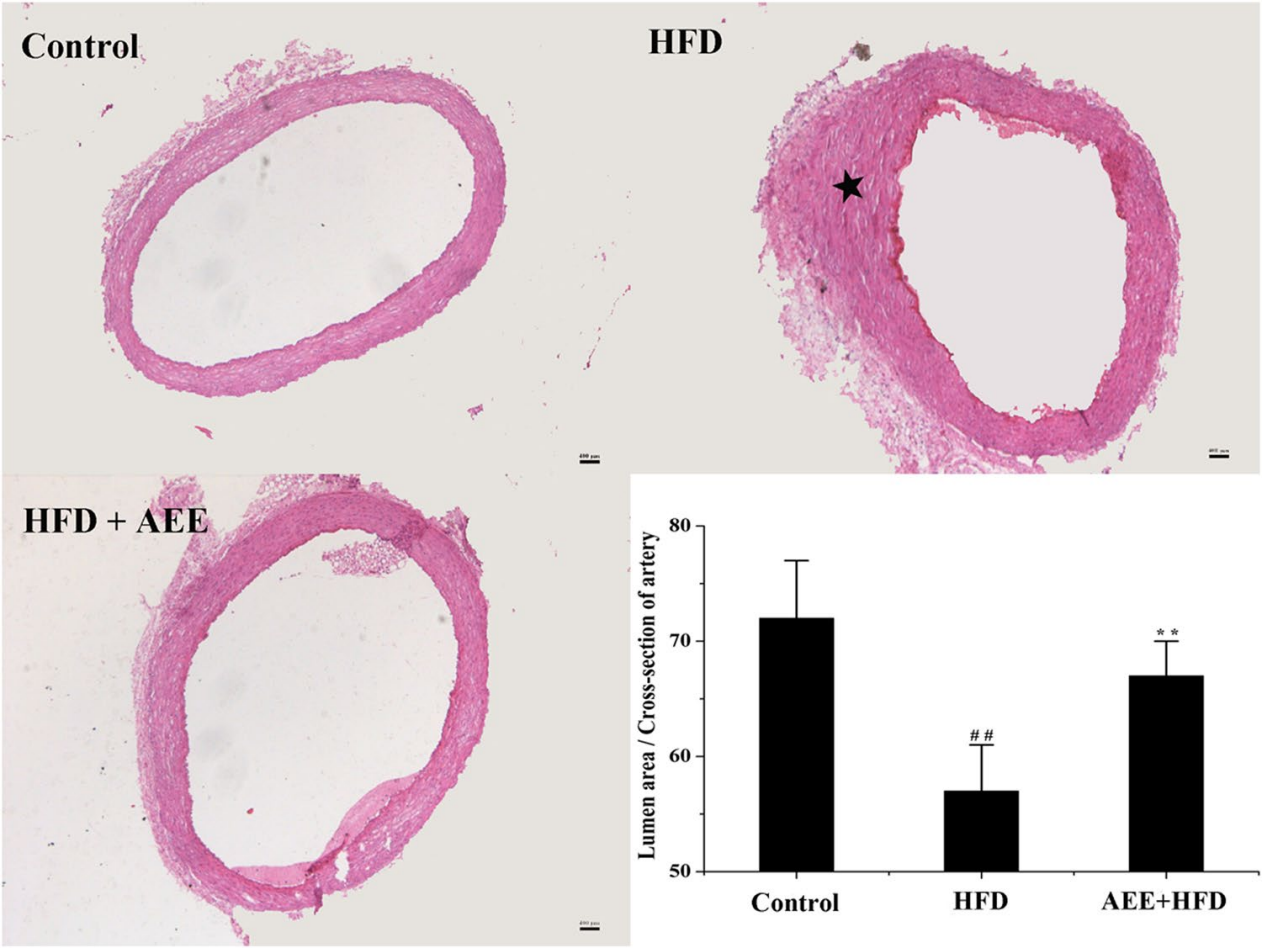
Representative cross-sections of aorta and image analysis of stenosis ratio in different group (×50). Compared with control group, blood vessel wall and lumina of aorta in HFD group became thicker and narrower (★). The stenosis ratio was expressed as percentage of lumen area to total area of the aorta. ^##^ *P* < 0.01 significant difference from the control; ^**^ *P* < 0.01 significant difference from HFD group.

No intimal or medial pathologic changes were observed in the aorta in control group (Fig. 3). Compared with the results in the control group, intimal thickening with foam cells and migration of smooth muscle cells were occurred in the HFD group. AEE caused a notable decrease of pathophysiologic changes of atherosclerosis induced by HFD. The percentage of lumen area to the cross-section of artery was also measured in the study. AEE treatment significantly ameliorated the aortic stenosis compared with the HFD group (Fig. 4, *P* < 0.01). The mean value of lumen area to artery cross-section in AEE group was lower than that in the control, but there was no statistical difference between two groups. These results demonstrated that anti-atherosclerosis effects of AEE might be associated with the improvement of the aorta lesion.

### Metabonomics analysis of plasma

**Figure 5:**
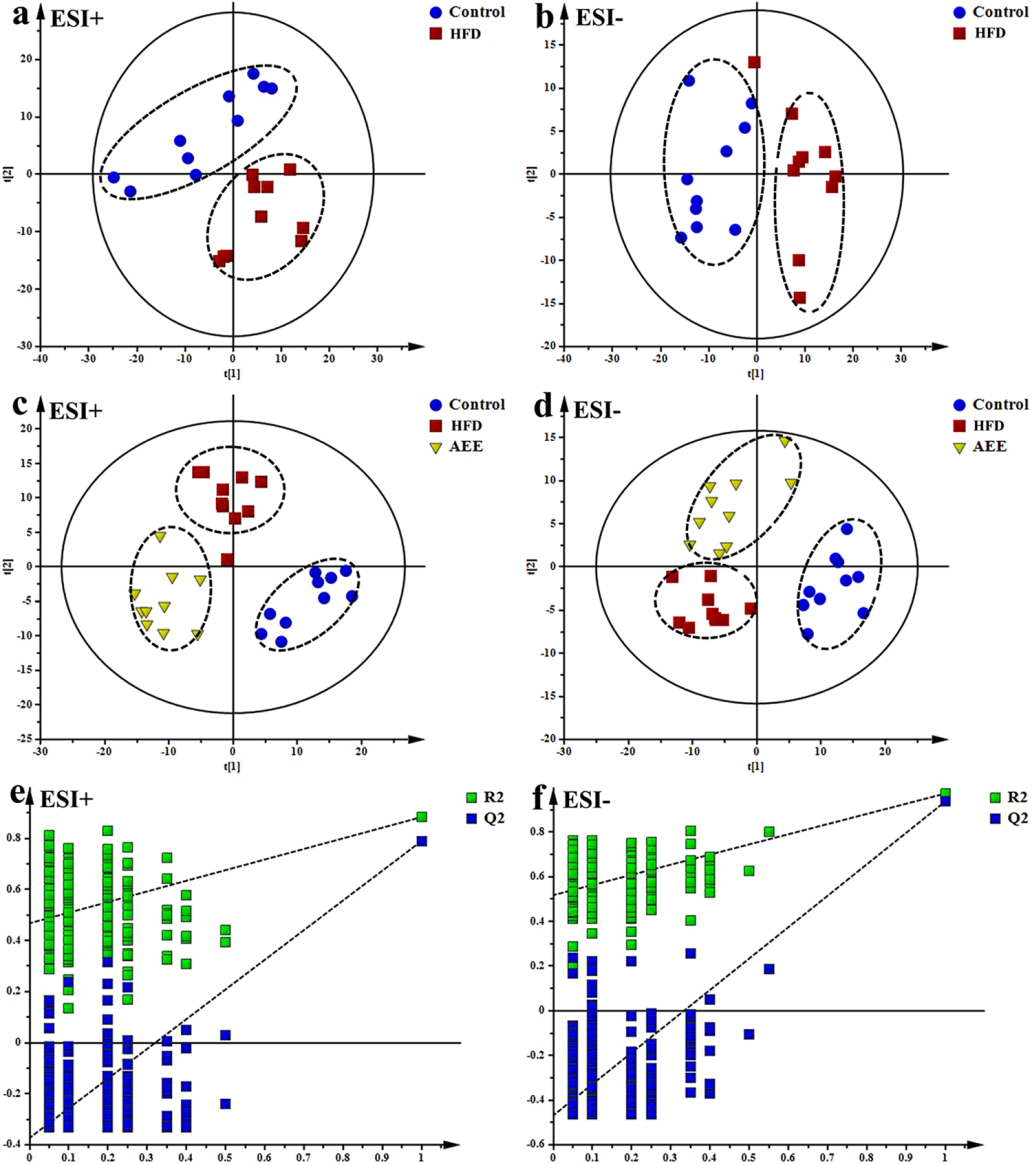
Multivariate data analyses of plasma based on UPLC-Q-TOF/MS analysis. (**a,b**) PCA score plot based on the plasma metabolic profiling of the control and atherosclerosis hamsters in positive and negative modes, ESI+: R^2^ = 0.627, ESI−: R^2^ = 0.625. (**c**,**d**) PLS-DA score plots of the control, HFD and AEE groups, ESI+: R^2^X = 0.424, R^2^Y = 0.911, Q^2^ = 0.813; ESI−: R^2^X = 0.613, R^2^Y = 0.917, Q^2^ = 0.829. (**e,f**) Permutation test of the PLS-DA models, ESI+: the intercepts of R^2^ = 0.468 and Q^2^ = −0.373, ESI−: R^2^ = 0.50, Q^2^ = −0.508.

In order to explore the possible action mechanisms of AEE, UPLC-Q-TOF/MS based metabonomic experiment was carried out. Representative total ion chromatograms (TICs) of the plasma samples analyzed by UPLC-Q-TOF/MS showed good separations and strong sensitivity of the established method (Fig. S1). PCA is an unsupervised multivariable statistical method to find out the metabolic distinction and the resulting data were displayed by score plots representing the distribution of samples in multivariate space. As indicated by the score plots in Fig. 5a and b, the plasma metabolic profiles in positive and negative modes of the control and HFD groups were clearly separated, which revealed that the perturbations of plasma metabolic profiles in HFD group were evident. Model parameter R^2^X representing the explanative ability of the model, were 0.627 and 0.625 in positive and negative modes, respectively, which showed the data can be highly elucidated by the two PCA models.

PLS-DA, a supervised multivariable statistical method, was conducted to further assess the influence of AEE on metabolic pattern. In PLS-DA analysis, the HFD and control groups were clearly separated, which was consistent with the found in PCA (Fig. 5c and d). Meanwhile, the metabolic profile of hamster in groups supplemented with AEE quite differed from the HFD group, indicating the disorders induced by HFD were ameliorated after AEE treatment. A clear separation among the control, HFD and AEE groups was observed in the score plots of the PLS-DA models. This distribution suggested that AEE treatments partially recovered the atherosclerosis status. The permutation test was performed to test the over-fitting of PLS-DA after modeling the data. Permutation tests generated the intercepts of R^2^ = 0.468 and Q^2^ = −0.373 in positive mode and R^2^ = 0.50, Q^2^ = −0.508 in negative mode (Fig. 5e and f), which demonstrated that the PLS-DA models were robust without overfitting.

VIP values concluding the contribution of the features for the model were employed to select the potential biomarkers. The candidate metabolites with VIP > 1.0 and *P* < 0.05 were considered as potential biomarkers. Following the threshold, 13 endogenous metabolites in plasma were selected, which may be related with how AEE influenced the development of atherosclerosis in hamster (Table 2). To fully and intuitively display the relationships and differences between samples, the selected biomarker data were analyzed using clustering heatmap (Fig. S2a). In the HFD group, the concentrations of lysophosphatidylcholine (LysoPC) (22:5), LysoPC (20:4), LysoPC (18:1), LysoPC (18:0), LysoPC (16:0), LysoPC (20:3), LysoPC (15:0), LysoPC (14:0), LysoPC (16:1) and elaidic acid were significantly increased, while leucine, valine and docosahexaenoic acid (DHA) were decreased (Table 2). Interestingly, AEE treatment corrected and reversed the variations of the selected potential biomarkers such as LysoPC (20:4), leucine and valine. These results indicated that AEE treatment had regulation effects on selected metabolites.

**Table 2:** Effects of AEE on potential biomarkers associated with atherosclerosis in plasma.

No.	RT	VIP	Formula	Metabolite	Adduct ion	m/z	Fold Change		Pathway
							HFD/Control	AEE/HFD	
1	9.97	5.94	C_24_H_50_NO_7_P	LysoPC (16:0)	[M+H]^+^	496.3401	1.14^*^	1.24^**^	Glycerophospholipid metabolism
2	10.36	5.74	C_26_H_52_NO_7_P	LysoPC (18:1)	[M+H]+	522.3560	2.07^**^	1.25^**^	Glycerophospholipid metabolism
3	12.15	4.01	C_26_H_54_NO_7_P	LysoPC (18:0)	[M+H]+	524.3718	1.22^*^	1.18^*^	Glycerophospholipid metabolism
4	10.06	2.01	C_30_H_52_NO_7_P	LysoPC (22:5)	[M+H]+	570.3559	5.59^**^	1.12	Glycerophospholipid metabolism
5	9.46	3.27	C_28_H_50_NO_7_P	LysoPC (20:4)	[M+H]+	544.3403	1.79^**^	1.15	Glycerophospholipid metabolism
6	1.48	1.86	C_6_H_13_NO_2_	Leucine	[M+H]+	132.1020	0.50^**^	1.68^**^	Amino acid metabolism
7	12.24	1.70	C_28_H_52_NO_7_P	LysoPC (20:3)	[M+H]+	546.3534	1.06	1.97	Glycerophospholipid metabolism
8	1.19	1.58	C_5_H_11_NO_2_	Valine	[M+H]+	118.0861	0.51^**^	2.22^*^	Amino acid metabolism
9	10.97	1.52	C_23_H_48_NO_7_P	LysoPC (15:0)	[M+H]+	482.3357	1.00	8.44^**^	Glycerophospholipid metabolism
10	8.52	1.44	C_22_H_46_NO_7_P	LysoPC (14:0)	[M+H]+	468.3090	8.54^**^	1.08	Glycerophospholipid metabolism
11	8.98	1.34	C_24_H_48_NO_7_P	LysoPC (16:1)	[M+H]+	494.3244	1.70^*^	1.26	Glycerophospholipid metabolism
12	12.28	1.54	C_22_H_32_O_2_	DHA	[M−H]−	327.2349	0.57^**^	0.75	Biosynthesis of unsaturated fatty acids
13	14.73	2.33	C_18_H_34_O_2_	Elaidic acid	[M−H]−	281.2506	1.75^**^	1.1	Biosynthesis of unsaturated fatty acids

DHA: docosahexaenoic acid; RT: retention time; LysoPC: lysophosphatidylcholine; ^*^ *P* < 0.05, ^**^ *P* < 0.01.

### Metabonomics analysis of urine

**Figure 6:**
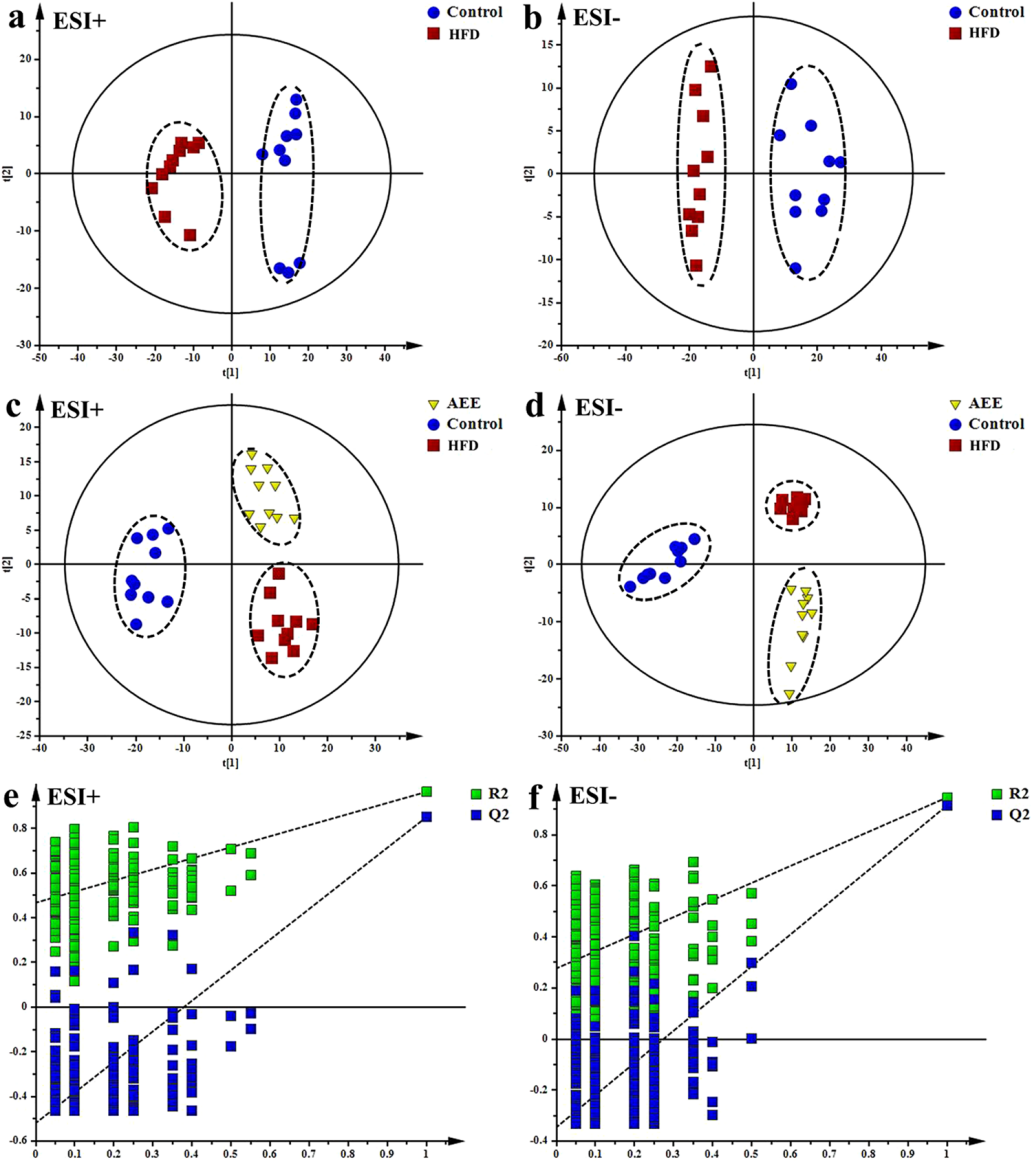
Multivariate data analyses of urine based on UPLC-Q-TOF/MS analysis. (**a**,**b**) PCA score plot based on the urine metabolic profiling of the control and atherosclerosis hamsters in positive and negative mode, ESI+: R^2^ = 0.535, ESI−: R^2^ = 0.75. (**c,d**) PLS-DA score plots of the control, HFD and AEE groups, ESI+: R^2^X = 0.614, R^2^Y = 0.966, Q^2^ = 0.884; ESI−: R^2^X = 0.7, R^2^Y = 0.96, Q^2^ = 0.932. (**e,f**) Permutation test of the PLS-DA models, ESI+: the intercepts of R^2^ = 0.468 and Q^2^ = −0.519, ESI−: R^2^ = 0.277, Q^2^ = −0.345.

The results of urine metabolomics were similar to that of the plasma samples, suggesting the ameliorative effects of AEE in hamster with atherosclerosis. Typical TICs of urine extracts in positive and negative modes obtained from UPLC-Q-TOF/MS analysis were shown in Fig. 3S. PCA was used to globally understand the metabolic changes of control and model groups. PCA score plots in positive and negative modes (Fig. 6a and b) showed that there were significant deviations in atherosclerotic hamsters compared with the control. The samples in model group clustered away from those in the control group, indicating HFD had significant influence on metabolites in urine.

Score plots of PLS-DA models showed a clear separation among control, model and AEE groups (Fig. 6c and d). The cluster of the samples in model group was located far away from the control, suggesting the urine metabolic profile of atherosclerosis was different from the healthy controls. Urine metabolic profile of hamsters in AEE treated group fairly differed from the model group, which indicated that AEE improved deviations induced by HFD. These results were in accordance with the results of blood lipids analysis and pathological changes observation. The validation plot (Fig. 6e and f) strongly indicated that the original model was valid: the Q^2^ regression line has a negative intercept (ESI+: Q^2^ = −0.519, ESI−: Q^2^ = −0.345), and all permuted R^2^ values to the left of the intercept (ESI+:R^2^ = 0.468, ESI−: R^2^ = 0.277) were lower than the original point to the right.

With VIP > 1.0 and *P* < 0.05, 17 metabolites were selected as potential biomarkers associated with atherosclerosis in urine (Table 3). Heatmap of the metabolites in urine was shown in Fig. S3b. In comparison with the control, HFD significantly elevated the levels of citric acid, phenylglucuronide, phenol sulphate, phenylacetylglycine, p-Cresol glucuronide and acetylcysteine, while HFD reduced the pantothenic acid, hippuric acid, phenyllactic acid, azelaic acid, niacinamide, spermidine, DL-2-Aminooctanoic acid (DL-2-AC), leucine and riboflavin. AEE treatment showed a tendency of bringing altered metabolites to normal, such as the improvement of citric acid, phenylglucuronide, phenol sulphate and acetylcysteine. Potential biomarkers in urine were mainly involved in citrate cycle, amino acid metabolism and nicotinate and nicotinamide metabolism.

**Table 3:** Effects of AEE on potential biomarkers associated with atherosclerosis in urine.

No.	RT	VIP	Formula	Metabolite	Adduction	m/z	Fold Change		Pathway
							HFD/Control	AEE/HFD	
1	1.16	1.30	C_6_H_8_O_7_	Citric acid	M−H	191.0191	5.23^**^	0.73	Citrate cycle
2	4.23	1.58	C_9_H_17_NO_5_	Pantothenic acid	M−H	218.1017	0.07^**^	0.74	beta-Alanine metabolism
3	6.05	2.88	C_12_H_14_O_7_	Phenylglucuronide	M−H	269.0654	4.09^**^	0.93	
4	6.29	2.54	C_6_H_6_O_4_S	Phenol sulphate	M−H	172.9899	2.54^**^	0.80^*^	
5	6.72	5.00	C_9_H_9_NO_3_	Hippuric acid	M−H	178.0494	0.29^**^	0.93	Phenylalanine metabolism
6	7.09	2.68	C_8_H_7_NO_4_S	Indoxyl sulfate	M−H	212.0007	0.77	0.81	Tryptophan metabolism
7	7.64	2.92	C_10_H_11_NO_3_	Phenylacetylglycine	M−H	192.0650	1.26^**^	0.88^*^	Phenylalanine metabolism
8	8.22	3.75	C_13_H_16_O_7_	p-Cresol glucuronide	M−H	283.0811	1.40^**^	0.88	
9	8.68	1.95	C_9_H_10_O_3_	Phenyllactic acid	M−H	165.0542	0.25^**^	0.98	Tropane, piperidine and pyridine alkaloid biosynthesi
10	9.89	1.09	C_9_H_16_O_4_	Azelaic acid	M−H	187.0960	0.38^**^	0.61^**^	
11	1.70	1.40	C_6_H_6_N_2_O	Niacinamide	M+H	123.0555	0.20^**^	0.57	Nicotinate and nicotinamide metabolism
12	0.88	1.04	C_7_H_19_N_3_	Spermidine	M+H	146.1652	0.33^**^	0.94	Arginine and proline metabolism
13	1.15	1.35	C_6_H_11_NO_2_	Pipecolic acid	M+H	130.0863	0.34	1.67	Lysine degradation
14	1.14	1.08	C_8_H_17_NO_2_	DL-2-AC	M+H	160.1331	0.27^**^	0.96	
15	1.87	1.26	C_6_H_13_NO_2_	Leucine	M+H	132.1018	0.30^*^	0.71	Valine, leucine and isoleucine degradation
16	7.26	1.22	C_17_H_20_N_4_O_6_	Riboflavin	M+H	377.1467	0.68^**^	0.51^**^	Vitamin digestion and absorption
17	8.74	1.21	C_5_H_9_NO_3_S	Acetylcysteine	M+H	164.0376	2.82^**^	0.86	

DL-2-AC: DL-2-Aminooctanoic acid, ^*^ *P* < 0.05, ^**^ *P* < 0.01.

### Pathway analysis

**Figure 7:**
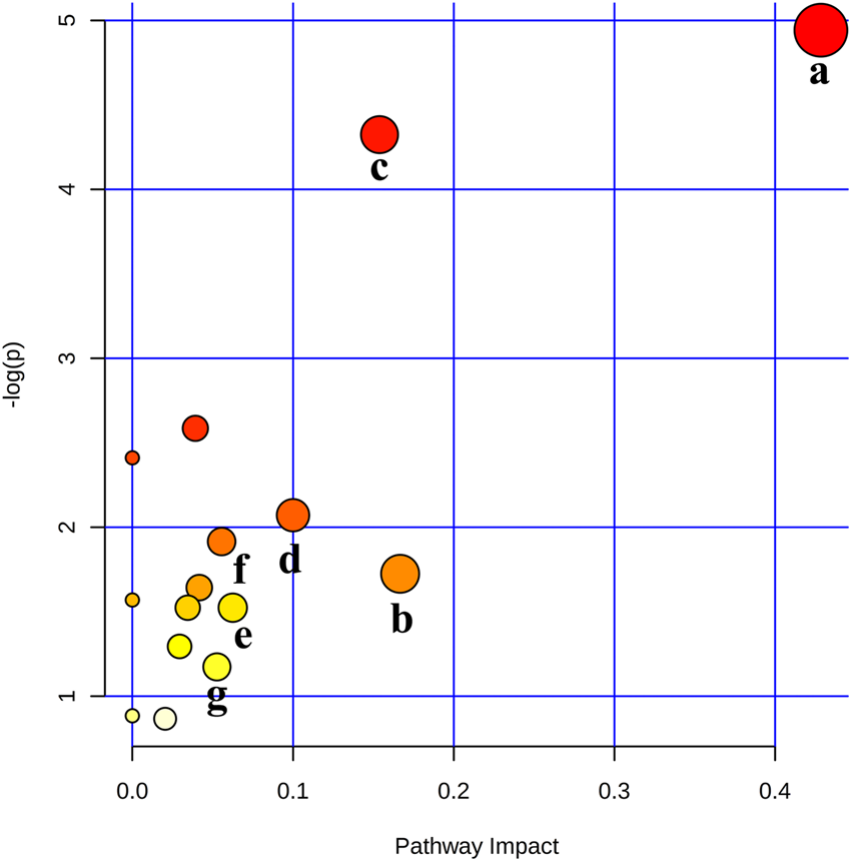
Possible disturbed metabolic pathways of the potential biomarkers for atherosclerosis and AEE treatment. (**a**) Valine, leucine and isoleucine biosynthesis; (**b**) Glyoxylate and dicarboxylate metabolism; (**c**) Pantothenate and CoA biosynthesis; (**d**) Riboflavin metabolism; (**e**) Lysine degradation; (**f**) Nicotinate and nicotinamide metabolism; (**g**) Glycerophospholipid metabolism.

Metabonomics pathway analysis was carried out with MetaboAnalyst 3.0 to identify and visualize the most relevant metabolic pathways in hamster with atherosclerosis. The impact-value threshold was set to 0.05 and the pathway with impact-value above this threshold was filtered out. The summary of pathway analysis was shown in Table S2. Figure 7 showed that the pathways in response to atherosclerosis and AEE treatment were valine, leucine and isoleucine biosynthesis, glyoxylate and dicarboxylate metabolism, pantothenate and CoA biosynthesis, riboflavin metabolism, lysine degradation, nicotinate and nicotinamide metabolism and glycerophospholipid metabolism. These pathways were obviously disturbed by HFD administration, and could be acted as targets for AEE against atherosclerosis.

## Discussion

Hamsters are high sensitive to HFD which can elevate blood lipid levels and promote appreciable atherosclerosis in as little as 6 weeks. Like humans, the cholesteryl ester transfer protein (CETP) of hamster can transfer the cholesterol from HDL to LDL particles in plasma. Therefore, HFD-fed hamster is an invaluable and sensitive model for rapid establishment of atherosclerosis^27^. To our knowledge, this study was the first to explore the anti-atherosclerosis effects and mechanism of AEE in atherosclerosis hamster model. The histopathological results confirmed that the hamsters suffered severe atherosclerotic lesions in the aorta and hepatic damages after administration of HFD for 12 weeks. Notably, the histopathological changes of aorta and liver were obviously improved after AEE treatment, which proved the therapeutic effects of AEE on atherosclerosis. Furthermore, biochemical analysis and UPLC-Q-TOF/MS based metabonomic were applied to characterize the crucial parameters and metabolic pathways associated with AEE treatment.

The analysis of biochemical parameters is helpful to assess the general health status of animals. It was reported that there was a close association between atherosclerosis and lipid abnormalities, especially high levels of plasma LDL, TCH, and TG^28^. In this study, the elevated levels of LDL, TCH, HDL and TG in HFD group showed the metabolic disorder of lipids, which was also verified by the metabolomics analysis in the score plots. AI was calculated to evaluate the lipid-lowering effect of AEE. Recent studies have suggested that AI is a reliable index to access the relative contribution of lipids to the atherosclerosis. The decreased AI values in AEE group revealed that AEE could ameliorate blood lipid profile, which was consistent with our previous study^23^. The normalization of blood lipid profile could contribute to reducing the accumulation of fat, lipid and cholesterol in the aorta, which might be the reasons for the improved pathological results in the aorta in AEE group. As the primary source of energy for the body, GLU is transported from the intestines or liver to cells via the bloodstream. High concentrations of GLU in the HFD group might be caused by the increased levels of blood lipids and energy metabolism disorders^29^. Serum TBIL, TP, ALB, ALT, AST and LDH are important parameters of liver function. The increased levels of these parameters in the HFD group might indicate existing liver damage, which was confirmed by the pathological changes of liver tissue. With the AEE treatment, hamsters showed a reversible trend to normal levels in biochemical parameters and had a remarkable decrease of pathological changes in liver. It was suggested that 12-week AEE treatment was beneficial to improve the biochemical profile and pathological changes in atherosclerosis hamster. Based these results, it could be found that there was a mutual cause-and-effect relationship between pathological findings and biochemical parameters.

Aspirin can reduce the risk of CVD by its anti-inflammatory and antiplatelet effects via the irreversible acetylation of cyclooxygenases (COX) 1 and 2^30^. COX-1 is constitutively expressed in most tissues, and is the predominant form in gastric mucosa which is crucial for mucosal protection. COX-2 is absent under normal conditions, but elevated levels are found during inflammation^31^. The non-selective inhibitation of aspirin on both COX-1 and COX-2 is considered to be an underlying reason for the gastrointestinal side effects. Accumulated evidence indicates that eugenol displays antiulcer activities, in which eugenol can dose-dependently reduce gastric ulcers in rat gastric ulcer models^32–34^. Eugenol can also stimulate the synthesis of mucus, an important gastroprotective factor, which may be responsible for antiulcer activity. AEE is decomposed into salicylic acid and eugenol by the enzyme after administration, then salicylic acid and eugenol can play complementary roles to reduce gastrointestinal damage. Meanwhile, there is no direct contact of acidic group with gastric mucosa through masking the carboxyl of aspirin, which is simple and efficient way to reduce gastrointestinal side effects^35^. In our previous study, no lesion in stomach and duodenum was found in the rats fed with 50 mg/kg AEE for 15 days^18^. However, slight pathological changes of gastric mucosa were observed in AEE group, which indicated there were some mild gastrointestinal side effects of AEE under the present experimental conditions. The pathological injury in gastric mucosa may be attributable to the animal species used in the experiment, long duration of the experiments (12 weeks) or the dosage of AEE (27 mg/kg). Chen Yu *et al*. reported that oral administration of aspirin (200 mg/kg) for seven days could induce severe mucosal damage in mice^36^. From the reasons of gastroprotective effects of eugenol and the disappearance of carboxyl group, it may be inferred that AEE has smaller gastrointestinal side effects than equal molar aspirin. Pathological change of gastric mucosa is a potential limitation in the application of AEE, which may be avoided by the appropriate control of dosage and administration time of AEE. Further studies are needed to reveal the underlying mechanism of gastrointestinal side effect in AEE treatment.

Metabolomics is a sensitive and effective approach for detecting biological responses by investigating the endogenous small molecule metabolites. Analysis of metabolite changes could provide valuable information, which is helpful to reveal the action mechanism of drug^37^. In this study, plasma and urine metabolomics analyses were applied to systematically evaluate the treatment effects of AEE in atherosclerosis hamsters. Multivariate data analysis indicated that the control group, HFD group, and AEE treatment group could be clearly distinguished from each other. Compared with the control, selected potential biomarkers associated with atherosclerosis exhibited differences in the HFD group, and be regulated by AEE treatment. Pathway analysis indicated that anti-atherosclerosis effects of AEE were mainly related with glycerophospholipid metabolism, amino acid metabolism, pantothenate and CoA biosynthesis, riboflavin metabolism, and biosynthesis of unsaturated fatty acids.

LysoPC is a major component of oxidized low-density lipoprotein, which plays functional roles in various diseases including diabetes, hyperlipidemia, atherosclerosis and cancer. It is generally believed that the increased LysoPCs can trigger inflammation and the autoimmune response, which may be related to the pathogenesis of atherosclerosis. In our study, LysoPCs were increased in hamster with HFD-induced atherosclerosis, which was good agreement with other reports^26^. The relative content of LysoPCs recovered at different levels after AEE administration except LysoPC (20:3), LysoPC (15:0) and LysoPC (16:1). It is noteworthy that there are still controversies on the roles of LysoPCs in atherosclerosis. For example, some researchers reported that some kinds of LysoPCs had a strong inverse association with coronary artery disease^38, 39^. After AEE treatment for 12 weeks, the abnormal levels of LysoPCs in atherosclerosis hamster were intervened. Perturbed glycerophospholipid metabolism suggested the complex physiological interplay during atherosclerosis progression. Further studies about the influence of AEE on LysoPCs are needed to elucidate its roles in atherosclerosis.

Diet supplementation with leucine not only plays key role in protein metabolism but also ameliorates lipid profile such as the reducing of TG, TCH and LDL. So the increased level of leucine in AEE group was conducive to lowing blood lipids and treating atherosclerosis. According to the results, it was found that there was some relationship among metabolite level changes, pathological results and biochemical parameters. Song *et al*. reported that elevated levels of GLU, lipid accumulation and decreased level of valine implied energy metabolism impairment such as glycolysis inhibitation and the increase of fatty acid β-oxidation^40^. In this study, the similar changes of GLU, blood lipids and valine were observed in HFD group. Citric acid, as a TCA cycle intermediate, was found to be higher in the HFD than that in the control. The increased free fatty acid oxidation and inhibitory TCA cycle might result in increasing the excretion of citric acid in urine^41^. Valine and leucine can be used to produce succinyl-CoA by degradation reaction, and then enter into TCA cycle. AEE might greatly promote TCA cycle and attenuate energy metabolism impairment by ameliorating blood lipid profile, reducing GLU and citric acid, as well as elevating the level of valine and leucine. Hippuric acid is glycine conjugate of benzoic acid, which was involved into phenylalanine metabolism. It has been report that urinary hippuric acid was decreased in atherosclerosis rabbits, which might be related to the dietary intake and gut microbial metabolism^42, 43^. Delaney J. *et al*. had reported that urinary phenylacetylglycine was raised in animals exhibiting abnormal phospholipid accumulation and might be a surrogate biomarker for phospholipidosis^44^. Increased phenylacetylglycine caused by HFD consumption was restored by AEE treatment, suggesting the improvement of phospholipid metabolism and the reduction of phospholipid accumulation.

Most studies have demonstrated the positive effects of dietary DHA on cardiovascular health, that is, DHA can reduce inflammation and total body fat and attenuate dyslipidemia^45^. Azelaic acid, a nine carbon saturated aliphatic dicarboxylic acid, can inhibit atherosclerosis development and exert beneficial effect on hepatic key enzymes of carbohydrate metabolism^46, 47^. In present study, we observed that the levels of DHA and azelaic acid in AEE group were lower than those in HFD group. The possible explanation for the results was that AEE increased the consumption of DHA and azelaic acid to produce inhibitory effect on inflammation and carbohydrate metabolism to against atherosclerosis development. Different studies have suggested a relationship between a high dietary intake of elaidic acid and increased risk of coronary artery disease through promoting lipid droplets accumulation^48^. HFD significantly increased the level of elaidic acid, which might be one of the reasons to explain the hepatic steatosis. Results showed that AEE treatment improved liver fat pathological changes, whereas no significant difference of elaidic acid was observed between HFD and AEE groups. It was speculated that AEE might have other therapeutic mechanism on the liver pathological changes induced by HFD.

Pantothenic acid is a B-group vitamin important for lipid metabolism and coenzyme A synthesis. Previous study has shown that HFD could decrease the pantothenic acid level in urine through the suppression of TCA cycle^49^. As a tryptophan metabolite, indoxyl sulfate is a circulating uremic toxin. It has been reported that indoxyl sulfate could stimulate glomerular sclerosis, increase systemic oxidative stress and induce endothelial dysfunction^50, 51^. In HFD group, the reduced excretion of indoxyl sulfate and pantothenic acid might be potential biomarkers of atherosclerosis, and that could accelerate the development of atherosclerosis. However, no significant difference of pantothenic acid and indoxyl sulfate was observed between HFD and AEE groups. Wei dong Dai *et al*. has reported that p-Cresol glucuronide and riboflavin are identified as potential biomarkers in endothelial dysfunction rats^52^. Like indoxyl sulfate, p-Cresol glucuronide is also a uremic toxin, which can inhibit endothelial cells proliferation and wounded endothelium repairment^53^. AEE treatment showed favorable inhibitation of p-Cresol glucuronide, indicating that anti-atherosclerosis efficacy of AEE might ascribe to the protection of endothelial cells. Riboflavin plays key role in maintaining health, which is required in many biological process such as energy production, red blood cell formation and reproduction. Moreover, riboflavin uptake can counteract oxidative stress and minimize cardiovascular risk^54^. Oxidative stress caused by HFD might increase riboflavin consumption to lead the reduction of riboflavin in the urine. AEE might aggravate riboflavin consumption to produce anti-atherosclerosis effects, resulting in further reduction of riboflavin in urine. Phenylglucuronide, phenol sulphate and acetylcysteine were increasingly excreted in the urines of the hamsters fed with HFD. They are usually served as antioxidants. In contrast to the healthy rats, increased phenylglucuronide, phenol sulphate and acetylcysteine in the urine of the atherosclerosis hamster suggested that more antioxidants were possibly produced to defense the increasing oxidative stress during pathological progression, and thereafter excreted massively in urine^55^. AEE treatment might inhibit the oxidative stress, and thus down-regulated levels of acetylcysteine and phenol sulphate. Niacinamide, spermidine and DL-2-AC were reduced in hamster with atherosclerosis, suggesting that there was a significant negative relationship between these metabolites and atherosclerosis. Very little is known about the mechanism of the reductions of these metabolites, and more studies are needed to explore their functional roles in atherosclerosis.

In the present work, the anti-atherosclerosis effect of AEE was confirmed by blood biochemistry, pathological examination and metabolomic analysis. Our results showed that AEE could significantly reduce HFD-induced body weight gains, normalize disturbed blood biochemistry, reduced excessive fat accumulation in hepatocyte and ameliorate pathological lesions of aorta. Furthermore, the PLS-DA score plots showed the complete distinction of HFD-induced atherosclerotic hamsters and AEE-treated hamsters. AEE effectively inhibited the metabolic alternations induced by atherosclerosis. Based on the metabonomic approach, the disturbed global metabolic profiling mainly associated with glycerophospholipid metabolism, amino acid metabolism, energy metabolism, riboflavin metabolism, and pantothenate and CoA biosynthesis was improved after AEE treatment. These findings demonstrated that UPLC-Q-TOF/MS-based metabonomic approach was a powerful tool to explore the underlying mechanism of atherosclerosis, and also be helpful for understanding the possible mechanism of AEE for anti-atherosclerosis.

## Electronic supplementary material

Supplementary information

## References

[1] Otsuka F, Yasuda S, Noguchi T, Ishibashi-Ueda H (2016). Pathology of coronary atherosclerosis and thrombosis. Cardiovasc Diagn Ther.

[2] Yogalakshmi B, Viswanathan P, Anuradha CV (2010). Investigation of antioxidant, anti-inflammatory and DNA-protective properties of eugenol in thioacetamide-induced liver injury in rats. Toxicology.

[3] Gill AO, Holley RA (2004). Mechanisms of bactericidal action of cinnamaldehyde against Listeria monocytogenes and of eugenol against L. monocytogenes and Lactobacillus sakei. Appl Environ Microbiol.

[4] Raghavendra RH, Naidu KA (2009). Spice active principles as the inhibitors of human platelet aggregation and thromboxane biosynthesis. Prostaglandins Leukot Essent Fatty Acids.

[5] Taher YA (2015). Experimental evaluation of anti-inflammatory, antinociceptive and antipyretic activities of clove oil in mice. Libyan J Med.

[6] Mnafgui K (2013). Inhibition of key enzymes related to diabetes and hypertension by Eugenol *in vitro* and in alloxan-induced diabetic rats. Arch Physiol Biochem.

[7] Venkadeswaran K (2014). Antihypercholesterolemic and Antioxidative Potential of an Extract of the Plant, Piper betle, and Its Active Constituent, Eugenol, in Triton WR-1339-Induced Hypercholesterolemia in Experimental Rats. Evid Based Complement Alternat Med.

[8] Venkadeswaran K, Thomas PA, Geraldine P (2016). An experimental evaluation of the anti-atherogenic potential of the plant, Piper betle, and its active constitutent, eugenol, in rats fed an atherogenic diet. Biomed Pharmacother.

[9] Vane JR, Botting RM (2003). The mechanism of action of aspirin. Thromb Res.

[10] Nansseu JR, Noubiap JJ (2015). Aspirin for primary prevention of cardiovascular disease. Thromb J.

[11] Sutcliffe P (2013). Aspirin in primary prevention of cardiovascular disease and cancer: a systematic review of the balance of evidence from reviews of randomized trials. PLoS One.

[12] Sorokin AV (2016). Addition of aspirin to a fish oil-rich diet decreases inflammation and atherosclerosis in ApoE-null mice. J Nutr Biochem.

[13] Yang, J. J. *et al*. Activation of activator protein 2 alpha by aspirin alleviates atherosclerotic plaque growth and instability *in vivo*. *Oncotarget* (2016). 10.18632/oncotarget.10400 PMC5288144 27391154

[14] Kean WF, Buchanan WW (2005). The use of NSAIDs in rheumatic disorders 2005: a global perspective. Inflammopharmacology.

[15] Li J-yong (2012). Synthesis of aspirin eugenol ester and its biological activity. Med Chem Res.

[16] Redasani VK, Bari SB (2012). Synthesis and evaluation of mutual prodrugs of ibuprofen with menthol, thymol and eugenol. Eur J Med Chem.

[17] Li J (2013). Genotoxic evaluation of aspirin eugenol ester using the Ames test and the mouse bone marrow micronucleus assay. Food Chem Toxicol.

[18] Li J (2012). A 15-day oral dose toxicity study of aspirin eugenol ester in Wistar rats. Food Chem Toxicol.

[19] Shen Y (2015). *In vivo* and *in vitro* metabolism of aspirin eugenol ester in dog by liquid chromatography tandem mass spectrometry. Biomed Chromatogr.

[20] Ma N (2015). Preventive Effect of Aspirin Eugenol Ester on Thrombosis in kappa-Carrageenan-Induced Rat Tail Thrombosis Model. PLoS One.

[21] Karam I (2016). Lowering effects of aspirin eugenol ester on blood lipids in rats with high fat diet. Lipids Health Dis.

[22] Dillard A, Matthan NR, Lichtenstein AH (2010). Use of hamster as a model to study diet-induced atherosclerosis. Nutr Metab (Lond).

[23] Karam I (2015). Regulation effect of Aspirin Eugenol Ester on blood lipids in Wistar rats with hyperlipidemia. BMC Vet Res.

[24] Wu Q (2014). UPLC-Q-TOF/MS based metabolomic profiling of serum and urine of hyperlipidemic rats induced by high fat diet. Journal of Pharmaceutical Analysis.

[25] Cardiff RD, Miller CH, Munn RJ (2014). Manual hematoxylin and eosin staining of mouse tissue sections. Cold Spring Harb Protoc.

[26] Liu Y-T (2014). UPLC-Q/TOF MS standardized Chinese formula Xin-Ke-Shu for the treatment of atherosclerosis in a rabbit model. Phytomedicine.

[27] Singhal AK, Finver-Sadowsky J, McSherry CK, Mosbach EH (1983). Effect of cholesterol and bile acids on the regulation of cholesterol metabolism in hamster. Biochim Biophys Acta.

[28] Rose H (2013). The effect of HIV infection on atherosclerosis and lipoprotein metabolism: A one year prospective study. Atherosclerosis.

[29] Akcilar R (2016). The effect of adropin on lipid and glucose metabolism in rats with hyperlipidemia. Iran J Basic Med Sci.

[30] Brune K, Hinz B (2004). The discovery and development of antiinflammatory drugs. Arthritis Rheum.

[31] Halter F, Tarnawski AS, Schmassmann A, Peskar BM (2001). Cyclooxygenase 2-implications on maintenance of gastric mucosal integrity and ulcer healing: controversial issues and perspectives. Gut.

[32] Santin JR (2011). Gastroprotective activity of essential oil of the Syzygium aromaticum and its major component eugenol in different animal models. Naunyn Schmiedebergs Arch Pharmacol.

[33] Capasso R, Pinto L, Vuotto ML, Di Carlo G (2000). Preventive effect of eugenol on PAF and ethanol-induced gastric mucosal damage. Fitoterapia.

[34] Garabadu D, Shah A, Singh S, Krishnamurthy S (2015). Protective effect of eugenol against restraint stress-induced gastrointestinal dysfunction: Potential use in irritable bowel syndrome. Pharm Biol.

[35] Redasani VK, Bari SB (2012). Synthesis and evaluation of mutual prodrugs of ibuprofen with menthol, thymol and eugenol. Eur J Med Chem.

[36] Yu C (2014). Effects of danshensu on platelet aggregation and thrombosis: *in vivo* arteriovenous shunt and venous thrombosis models in rats. PLoS One.

[37] Fillet M, Frederich M (2015). The emergence of metabolomics as a key discipline in the drug discovery process. Drug Discov Today Technol.

[38] Ganna A (2014). Large-scale metabolomic profiling identifies novel biomarkers for incident coronary heart disease. PLoS Genet.

[39] Basak T (2015). Identification of metabolic markers in coronary artery disease using an untargeted LC-MS based metabolomic approach. J Proteomics.

[40] Song X (2013). (1)H NMR-based metabolomics approach to evaluate the effect of Xue-Fu-Zhu-Yu decoction on hyperlipidemia rats induced by high-fat diet. J Pharm Biomed Anal.

[41] Kim SH (2009). 1H-nuclear magnetic resonance spectroscopy-based metabolic assessment in a rat model of obesity induced by a high-fat diet. Anal Bioanal Chem.

[42] Hasegawa M (2007). Urinary metabolic fingerprinting for amiodarone-induced phospholipidosis in rats using FT-ICR MS. Exp Toxicol Pathol.

[43] Liu Y-T (2014). Urinary metabonomic evaluation of the therapeutic effect of traditional Chinese medicine Xin-Ke-Shu against atherosclerosis rabbits using UPLC–Q/TOF MS. Chemometr Intell Lab.

[44] Delaney J (2004). Phenylacetylglycine, a putative biomarker of phospholipidosis: its origins and relevance to phospholipid accumulation using amiodarone treated rats as a model. Biomarkers.

[45] Poudyal H, Panchal SK, Ward LC, Brown L (2013). Effects of ALA, EPA and DHA in high-carbohydrate, high-fat diet-induced metabolic syndrome in rats. J Nutr Biochem.

[46] Muthulakshmi S, Saravanan R (2013). Efficacy of azelaic acid on hepatic key enzymes of carbohydrate metabolism in high fat diet induced type 2 diabetic mice. Biochimie.

[47] Litvinov D (2010). Anti-atherosclerotic actions of azelaic acid, an end product of linoleic acid peroxidation, in mice. Atherosclerosis.

[48] Oomen CM (2001). Association between trans fatty acid intake and 10-year risk of coronary heart disease in the Zutphen Elderly Study: a prospective population-based study. The Lancet.

[49] Zhu Y (2013). Effect of metformin on the urinary metabolites of diet-induced-obese mice studied by ultra performance liquid chromatography coupled to time-of-flight mass spectrometry (UPLC-TOF/MS). J Chromatogr B Analyt Technol Biomed Life Sci.

[50] Tumur Z, Niwa T (2009). Indoxyl sulfate inhibits nitric oxide production and cell viability by inducing oxidative stress in vascular endothelial cells. Am J Nephrol.

[51] Niwa T, Ise M (1994). Indoxyl sulfate, a circulating uremic toxin, stimulates the progression of glomerular sclerosis. J Lab Clin Med.

[52] Dai W (2011). Effect of the traditional Chinese medicine tongxinluo on endothelial dysfunction rats studied by using urinary metabonomics based on liquid chromatography-mass spectrometry. J Pharm Biomed Anal.

[53] Dou L (2004). The uremic solutes p-cresol and indoxyl sulfate inhibit endothelial proliferation and wound repair. Kidney Int.

[54] Apelt N (2009). ACP1 genotype, glutathione reductase activity, and riboflavin uptake affect cardiovascular risk in the obese. Metabolism.

[55] Gao X (2012). Systematic variations associated with renal disease uncovered by parallel metabolomics of urine and serum. BMC Syst Biol.

