# Supplementary material for: UPLC-Q-TOF/MS-based metabonomic studies on the intervention effects of aspirin eugenol ester in atherosclerosis hamsters
Source: Sci Rep. 2017 Sep 5;7:10544. doi: 10.1038/s41598-017-11422-7 (PMC5585262; doi:10.1038/s41598-017-11422-7)
Supplement: Supplementary file 1 — Supplementary information [file 41598_2017_11422_MOESM1_ESM.pdf]

UPLC-Q-TOF/MS-based metabonomic studies on the intervention effects of aspirin eugenol ester in atherosclerosis hamsters

Ning Ma, Yajun Yang, Xiwang Liu, Xiaojun Kong, Shihong Li,

Zhe Qin, Zenghua Jiao, Jianyong Li*

Key Lab of New Animal Drug Project of Gansu Province, Key Lab of Veterinary Pharmaceutical Development, Ministry of Agriculture, Lanzhou Institute of Husbandry and Pharmaceutical Science of Chinese Academy of Agricultural Sciences, Lanzhou, China

*Corresponding author:

Name: Jian-Yong Li

Address: No.335, Jiangouyan, Qilihe district, Lanzhou 730050, China

E-mail: lijy1971@163.com

Telephone: 0086-0931-2115290

Fax: 0086-0931-2115290

Table S1 Optimized gradient elution program of UPLC-Q-TOF/MS in plasma and urine metabonomic analysis.

Time (min)	Plasma		Time (min)	Urine	
	Solvent A (%)	Solvent B (%)		Solvent A (%)	Solvent B (%)
0	95	5	0	98	2
3	60	40	0.5	98	2
7	40	60	7	80	20
9	15	85	9	65	35
16	5	95	13	30	70
20	5	95	15	2	98
			17	2	98
			19	98	2
			20	98	2

Solvent A: water with 0.1% formic acid (by volume); Solvent B: acetonitrile with 0.1% formic acid (by volume).

Table S2 Pathway analysis result with MetaboAnalyst 3.0 of the potential biomarkers in plasma and urine.

No.	Pathway name	Total	Expected	Hits	Raw p	-log(*P*)	Impact
1	Valine, leucine and isoleucine biosynthesis	11	0.13338	2	0.007141	4.9419	0.42857
2	Glyoxylate and dicarboxylate metabolism	16	0.19401	1	0.1782	1.7249	0.16667
3	Pantothenate and CoA biosynthesis	15	0.18188	2	0.013249	4.3238	0.15384
4	Riboflavin metabolism	11	0.13338	1	0.126	2.0714	0.1
5	Lysine degradation	20	0.24251	1	0.21782	1.5241	0.0625
6	Nicotinate and nicotinamide metabolism	13	0.15763	1	0.14724	1.9157	0.05556
7	Glycerophospholipid metabolism	30	0.36377	1	0.30916	1.1739	0.05263
8	Aminoacyl-tRNA biosynthesis	67	0.81241	2	0.19338	1.6431	0.04166
9	Valine, leucine and isoleucine degradation	38	0.46077	2	0.075344	2.5857	0.03922
10	Citrate cycle (TCA cycle)	20	0.24251	1	0.21782	1.5241	0.03448
11	Glutathione metabolism	26	0.31526	1	0.2739	1.295	0.02941
12	Arginine and proline metabolism	44	0.53352	1	0.4203	0.86679	0.02041
13	Biosynthesis of unsaturated fatty acids	42	0.50927	2	0.08971	2.4112	0
14	beta-Alanine metabolism	19	0.23039	1	0.20809	1.5698	0
15	Fatty acid biosynthesis	43	0.5214	1	0.41296	0.88441	0

Total: The total number of compounds in the pathways; the hits are the actually matched number from the user upload data; the raw p is the original p value calculated from the enrichment analysis; the impact is the pathway impact value calculated from pathway analysis.

Figure S1 Representative TICs of the plasma samples analyzed by UPLC-Q-TOF in positive and negative modes.

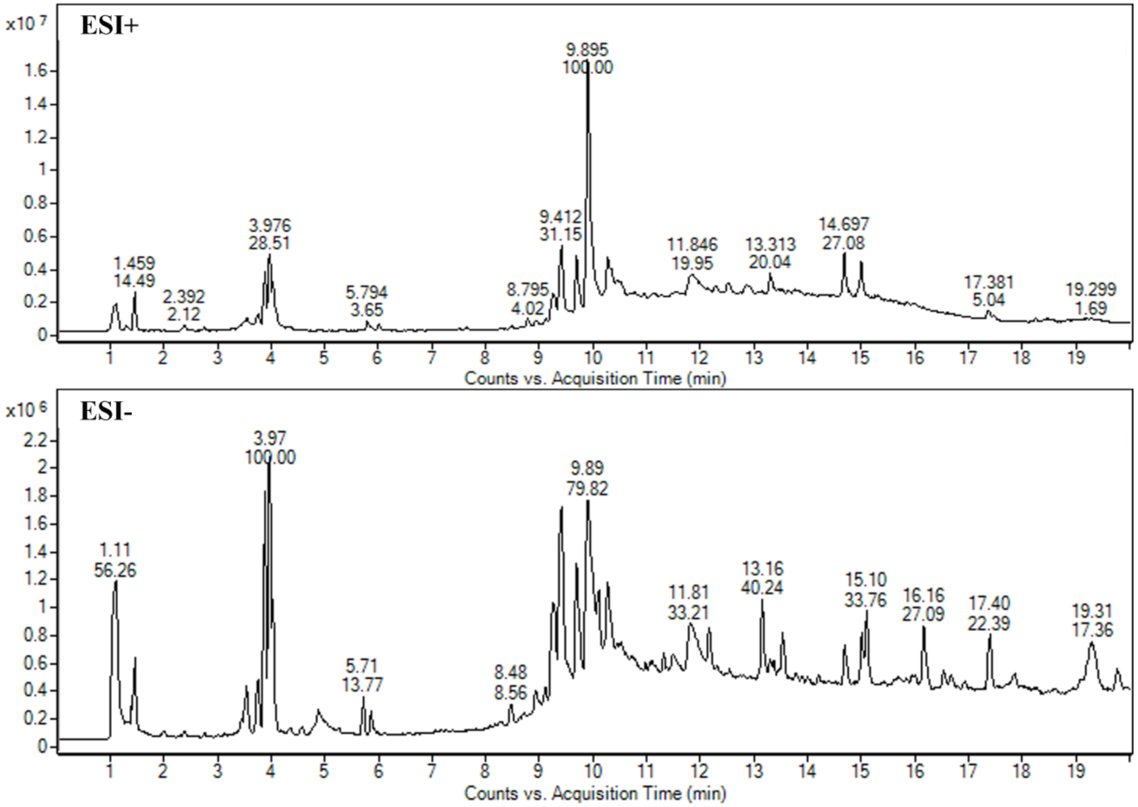

Retention time and abundance percentage were labeled upon the peaks.

Fig. S2 Clustering heatmap of potential biomarkers in plasma associated with atherosclerosis and AEE treatment.

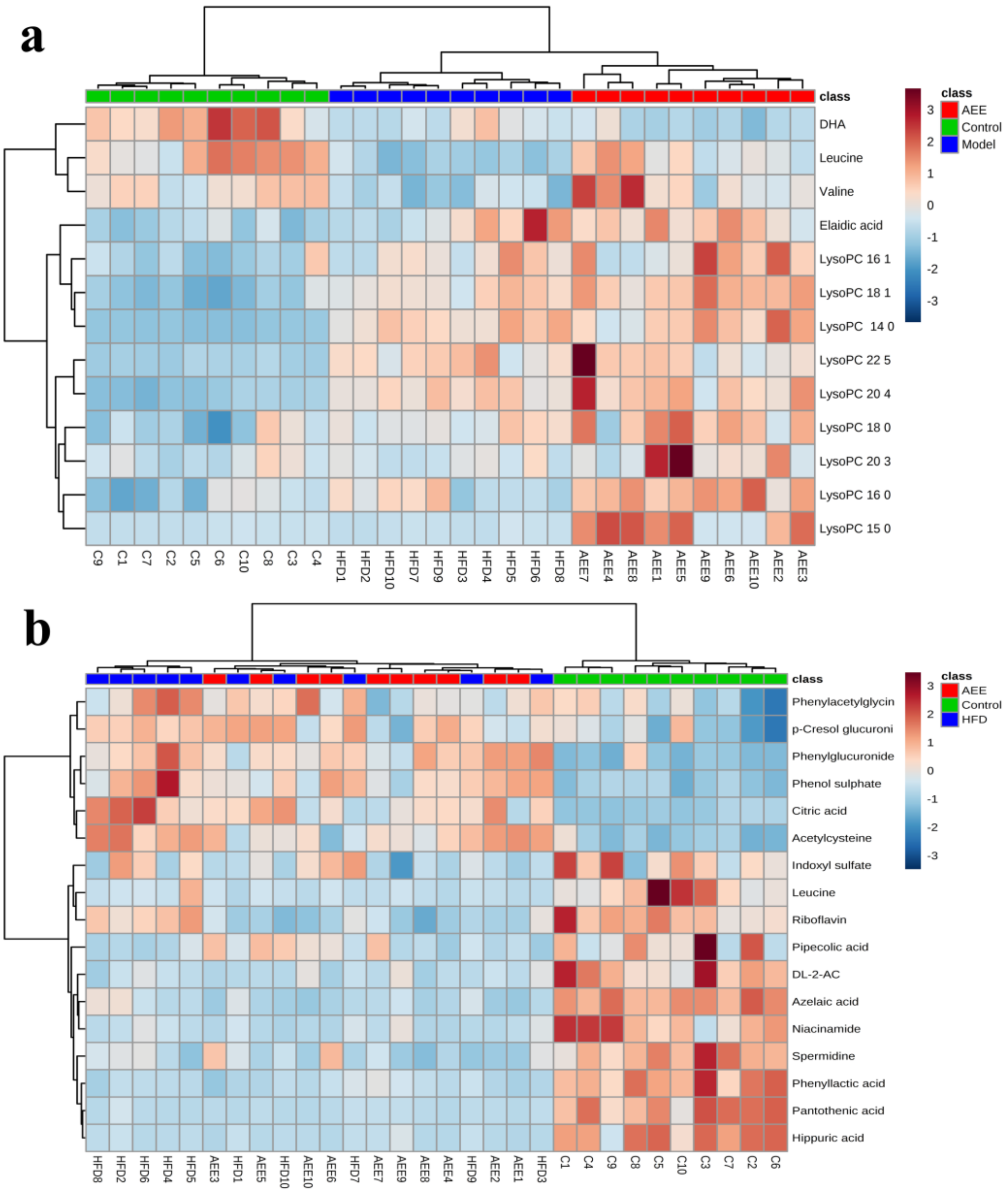

Differences of samples and metabolites were hierarchically clustered. Increasing expression values were coded with green to red colors. Rows indicated potential biomarkers; columns indicated samples.

Figure S3 Representative TICs of the urine samples analyzed by UPLC-Q-TOF in positive and negative modes.

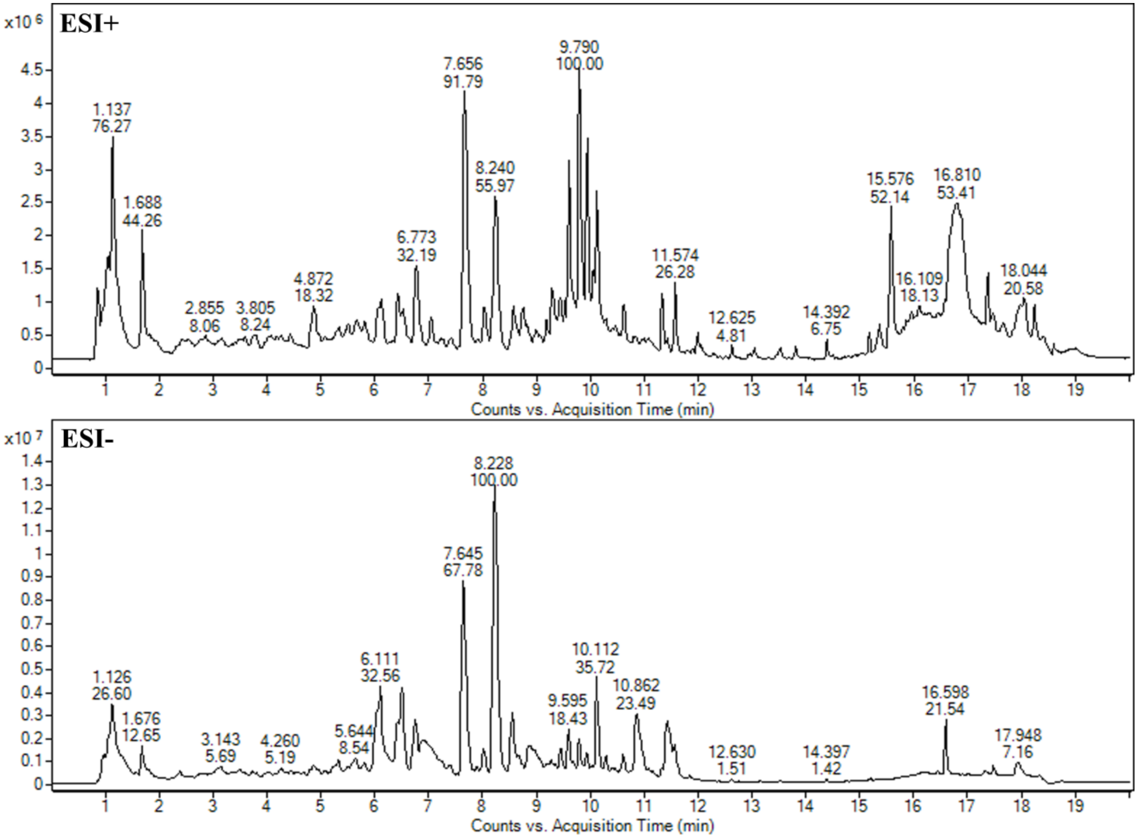

Retention time and abundance percentage were labeled upon the peaks.
